# AB thymoma revealed by a huge intraparenchymal lung mass: a case report

**DOI:** 10.11604/pamj.2021.38.189.28041

**Published:** 2021-02-18

**Authors:** Btissame Es-sabbahi, Mounia Serraj, Baderdine Alami, Mohammed Elbiaze, Mohammed Chakib Benjelloun, Bouchra Amara

**Affiliations:** 1 Faculty of Medicine and Pharmacy, Sidi Mohammed Ben Abdellah University, Fes, Morocco,; 2 Department of Pneumology, University Hospital Center Hassan II, Fes, Morocco,; 3 Department of Radiology, University Hospital Center Hassan II, Fes, Morocco

**Keywords:** Thymoma, ectopics locations, primary intrapulmonary thymoma, case report

## Abstract

Thymoma is an epithelial neoplasm of the thymus, which commonly lies in the anterior mediastinum. Unusually it can be found in other locations as well. Ectopic thymoma rarely presents as an intrathoracic tumor. We report a case of ectopic thymoma presenting as a giant right intrathoracic tumor, the patient was 51-year-old, and who was presented with heaviness in chest and breathlessness. Detailed investigation including chest computed tomography scan revealed a well-defined large solid tumor in the right thoracic cavity, in this case, immunohistochemical analysis demonstrated a thymome AB. The tumor was metastatic to the lung. Patient received a neoadjuvant chemotherapy, with favorable evolution.

## Introduction

Thymic epithelial tumors are rare tumors with variable evolution and prognosis. These are the most frequent tumors of the anterior mediastinum [1,2]. These tumors develop in more of 90% of cases in the anterior mediastinum [3], and diagnosis is made in 2/3 of cases on the incidental discovery of a mass in the anterior mediastinum [3]. However, ectopic thymoma has been reported in a variety of sites [4,5], such as the neck (ectopic cervical thymoma, ECT) [6,7], middle mediastinum [8], posterior mediastinum, pleura [9], lung [10] and the heart. We report the case of an AB thymoma presenting as a giant intrathoracic tumor.

## Patient and observation

**Figure 1:**
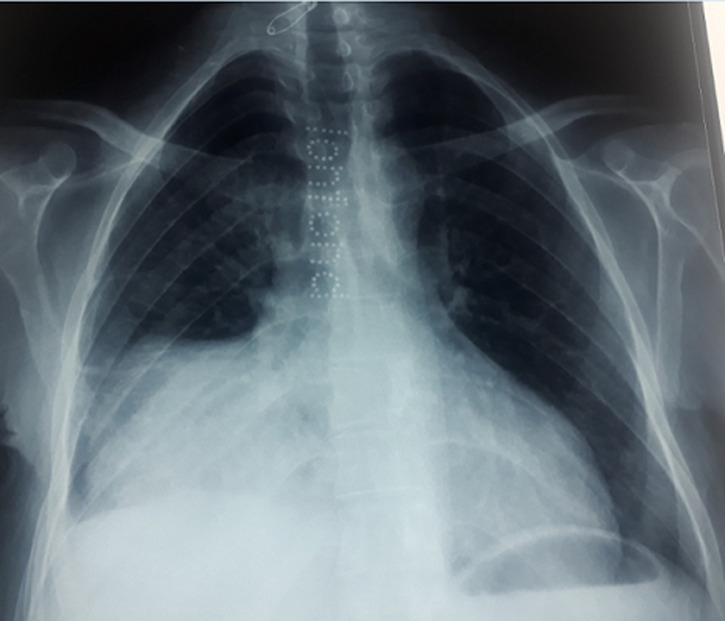
homogenous lesion in the right hemithorax extending from the hilum to the level of diaphragmatic dome; the medial border merged with the mediastinum with loss of the cardiac silhouette

**Figure 2:**
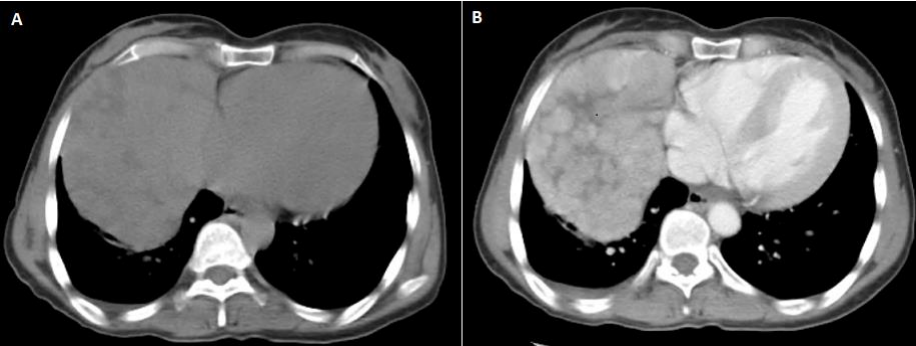
A) non-contrast CT chest and B) contrast-enhanced CT chest: heterogeneously enhancing mass in the right thoracic cavity; it showed a clear limits with polycyclic contours, and it occupies the middle lobe and extends to the upper and lower lobes

A 51-year-old patient, with a 5-month history off dyspnea, at the physical examination a right basithoracic condensation syndrome was found, radiograph of the chest showed well-defined homogenous lesion in the right hemithorax extending from the hilum to the level of diaphragmatique dome. The medial border merged with the mediastinum with loss of the cardiac silhouette. There was no air bronchogram, calcification, cavitation or rib destruction (Figure 1). Chest computed tomography (Figure 2) showed a solid tumor measuring 120x90x80cm in the right thoracic cavity. The tumor showed a clear limits with polycyclic contours, it occupies the middle lobe and extends to the upper and lower lobes via the splits which have a discontinuous appearance, it comes into contact with the right cardiac cavities without any fatty separation border, with presence of secondary straight pulmonary nodules. Bronchoscopy objectified an aspect of extrinsic compression on the basal pyramid. A primitive bronchial cancer was evoked, as well as a solitary fibrous tumour (polylobate aspect of the mass). The tumour was already metastatic in the lung. An echoguided biopsy of this mass was performed, the anatomopathological examination concluded to a thymoma of type AB, with a positive CD5 and terminal deoxynucleotidyl transferase (TDT). She received chemotherapy according to the College of America Pathologists (CAP) protocol (cisplatin, doxorubicin, cyclophosphamide), a control CT scan after 3 cures showed a discrete decrease in size of the mass, with disappearance of secondary pulmonary nodules, a surgical procedure will be discussed.

## Discussion

The thymus is located in the upper front part of the chest, in the anterior superior mediastinum, behind the sternum, and infront of the heart. The thymus continues to grow after birth, until it reaches its maximum weight at puberty 20-50 grams. After puberty, the thymus becomes progressively involved. In the adult, the parenchyma is gradually replaced by fatty tissue without disappearing completely. Thymoma is the most common primary mediastinal neoplasm in adults and the most frequent tumor of the anterior mediastinum, originating from the epithelial cells of the thymus. Ninety six percent (96%) of the tumors occurring in the anterior or anterosuperior mediastinum, but ectopic localizations have been described in the literature, mainly involving the neck, middle mediastinum, posterior mediastinum, pleura, lung [4-9]. Primary intrapulmonary thymomas (PIT) are very rare events, and are defined as intrapulmonary tumors without associated mediastinal localization, whose anatomopathological examination reveals a thymoma [11]. There is still a great deal of debate about the pathogenesis of PIT, the most widespread theory being embryologic displacement. The human embryo has five pairs of entobranchial pockets whose entoblastic coating gives rise to different organs. The thymus is derived from the third entoblastic pharyngeal sac. In the 5^th^ week of development, the two thymic blanks appear. Then before the 7^th^ week, they migrate in caudal and medial positions to end up under the thyroid gland in a retro sternal intra thoracic position. Accidently, some islands of thymus tissue may be left in unrelated anatomical regions due to defects during descending of thymic buds. This residual tissue may be the cause of developing some disorders such as thymic cyst, cervical thymoma, and ectopic thymus tissue [12]. Although critics of this theory point out that the respiratory primordium develops several weeks before descent of the thymic primordium [11,13]. The second hypothesis is that thymomas can develop from pluripotent stem cells capable of differentiating and giving different histological types. This theory is supported by the unusual localization of other tumors in the lung, namely meningiomas, salivary gland tumors [11,14].

Cases of PIT are very rare, about thirty observations of primary intra pulmonary thymoma have been published since 1951 [11,15-20]. Most of the cases described in the literature occurred after the 5^th^ decade of life, so for our patient she was 51 years old [11]. A slight female predominance was reported, with a much more frequent localization in the right lung [11]. And this was the case of our patient, whose tumor is located in the middle lobes, and extends to the upper and lower lobes. The majority of these tumors have been discovered on chest X-ray in asynmptomatic patients [11,13]. Presenting symptoms have included cough [11], chest pain, asthenia, hemoptysis [13]. In the case described in the present study, the patient had continuous dyspnea, hence the realization of a chest X-ray, which showed a voluminous right paracardiac mass. A primitive bronchopulmonary cancer was strongly suspected, a solitary fibrous tumor was also evoked because off the polylobate character ofthe mass, a bronchial fibroscopy was performed, which found an aspect of extrinsic compression at the level of the basal pyramid, without detectable endobronchial lesions, and since the tumor was already metastatic, surgical resection was not possible, an echoguided trans parietal biopsy was performed. These tumors have a better prognosis when it is surgically resected [11,20]. For our patient, the tumor was already metastatic in the lung, she received neoadjuvant chemotherapy, with favorable evolution, after 3 cures of chemotherapy according to the CAP protocol a regression of the volume of the tumor mass was noted, with disappearance of the secondary pulmonary localizations, a surgical procedure will be discussed.

## Conclusion

Ectopic thymomas, even if it is a very rare event, should always be included in the differential diagnosis whenever a large intra thoracic mass is observed, and the histopathological aspect is not typical of a primitive bronchopulmonary.
